# Xanthogranulomatous Cholecystitis in a 15-Year-Old Girl: A Case Report and Literature Review

**DOI:** 10.7759/cureus.78205

**Published:** 2025-01-29

**Authors:** William Qian, Nestor Sabat, Ishanth D Jayewardene

**Affiliations:** 1 General Surgery, Royal North Shore Hospital, St Leonards, AUS; 2 Medicine and Health, The University of Sydney, Camperdown, AUS; 3 General Surgery, Port Macquarie Base Hospital, Port Macquarie, AUS; 4 Science and Health, School of Rural Medicine, Charles Sturt University, Port Macquarie, AUS; 5 Medicine and Health, The University of New South Wales, Kensington, AUS

**Keywords:** adolescent, bile leak, cholecystitis, endoscopic retrograde cholangiopancreatography, general surgery, laparoscopic cholecystectomy, pediatric cholecystitis, pediatric surgery, subtotal cholecystectomy, xanthogranulomatous cholecystitis

## Abstract

Xanthogranulomatous cholecystitis (XGC) is a rare and severe variant of cholecystitis that poses significant diagnostic and surgical challenges. While predominantly seen in adults, its occurrence in pediatric patients is exceedingly rare, with very few cases documented in the literature. We present the case of a 15-year-old girl initially diagnosed with acute calculous cholecystitis who underwent a laparoscopic cholecystectomy. Intraoperatively, extensive pseudo-tumorous inflammation was identified, with fusion of the gallbladder into the omentum, duodenum, and colon, necessitating a subtotal cholecystectomy. Her postoperative recovery was complex, requiring endoscopic retrograde cholangiopancreatography (ERCP), and she was ultimately discharged after a prolonged hospital stay. Histopathological examination confirmed the diagnosis of XGC. This case highlights the complexities of managing this rare entity in pediatric patients.

## Introduction

Xanthogranulomatous cholecystitis (XGC) is a rare and severe chronic inflammatory condition of the gallbladder wall [1], characterized by the presence of lipid-laden macrophages, fibrosis, and destructive inflammation [2,3]. XGC often mimics gallbladder carcinoma due to its pseudo-tumorous nature [4], with extensive fibrosis and wall thickening that may involve surrounding structures such as the liver, duodenum, and colon [5]. While it is most often seen in adults over the age of 50 [6], pediatric cases are exceedingly rare, with very few reports in the literature documenting its occurrence in children or adolescents. The pathogenesis of XGC in children remains incompletely understood, although in adults biliary obstruction and chronic inflammation are believed to play key roles [4]. The preoperative diagnosis of XGC remains challenging despite advances in imaging, and its pseudo-tumorous nature often leads to diagnostic dilemmas [7]. Given its rarity in pediatric patients, there is a paucity of literature on the optimal management of XGC in this population. We herein discuss a rare case of XGC in a 15-year-old girl that highlights the diagnostic and surgical challenges posed by this rare entity. This case underscores the importance of recognizing XGC as a differential diagnosis in pediatric patients presenting with complex gallbladder disease.

## Case presentation

**Figure 1:**
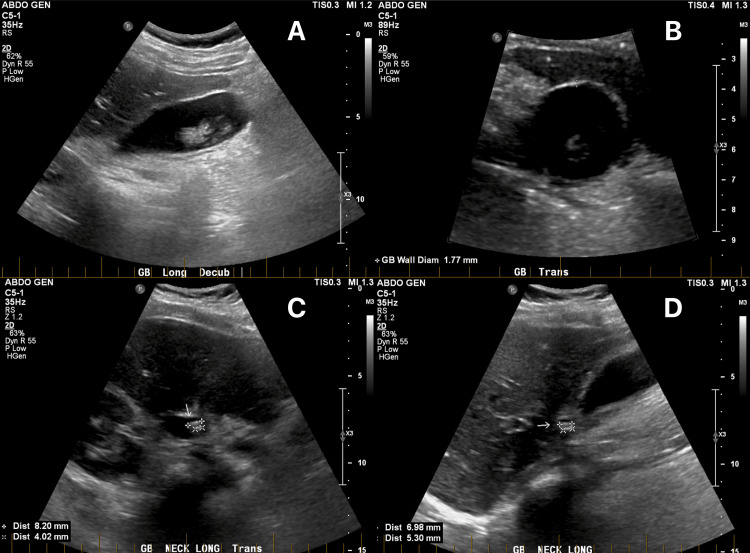
Ultrasound of the gallbladder on initial presentation. A: Longitudinal view. B: Thin gallbladder wall with a diameter of 1.77 mm. C, D: Gallstones impacted in the gallbladder neck.

A 15-year-old girl presented to the emergency department with 24 hours of right upper quadrant abdominal pain associated with multiple episodes of vomiting and fevers. This was on a background of perforated, gangrenous appendicitis that was managed with laparoscopic appendicectomy eight months prior. There was no other significant medical history. On examination, she was febrile to 38.0°C but hemodynamically stable. Her abdomen was tender in the right upper quadrant and Murphy’s sign was positive. Routine biochemistry revealed elevated inflammatory markers but normal liver function tests (Table 1). An ultrasound of the gallbladder demonstrated multiple gallstones, the largest measuring 8 mm, with two impacted in the gallbladder neck (Figure 1). There were otherwise no features of cholecystitis, with a thin gallbladder wall measuring 1.77 mm. A diagnosis of early calculous cholecystitis was made, and the patient was admitted for treatment with intravenous ampicillin and gentamicin. The following day, her pain had markedly improved, and she was discharged home with a course of oral amoxicillin-clavulanic acid and a plan to return for an elective laparoscopic cholecystectomy in four weeks.

**Table 1:** Relevant blood test results on the initial and second emergency presentations.

Pathology	Initial presentation	Second presentation	Normal range
Bilirubin	15 umol/L	7 umol/L	<20 umol/L
Alkaline phosphatase	93 unit/L	93 unit/L	30 - 110 unit/L
Gamma-glutamyltransferase	10 unit/L	22 unit/L	5 - 50 unit/L
Alanine aminotransferase	<6 unit/L	13 unit/L	1 - 40 unit/L
Aspartate aminotransferase	15 unit/L	34 unit/L	10 - 35 unit/L
C-reactive protein	250 mg/L	223 mg/L	<5 mg/L
White blood cell count	25.5 x 10^9^/L	14.8 x 10^9^/L	4.0 - 11.0 x 10^9^/L
Neutrophils	21.6 x 10^9^/L	11.0 x 10^9^/L	2.0 - 8.0 x 10^9^/L

**Figure 2:**
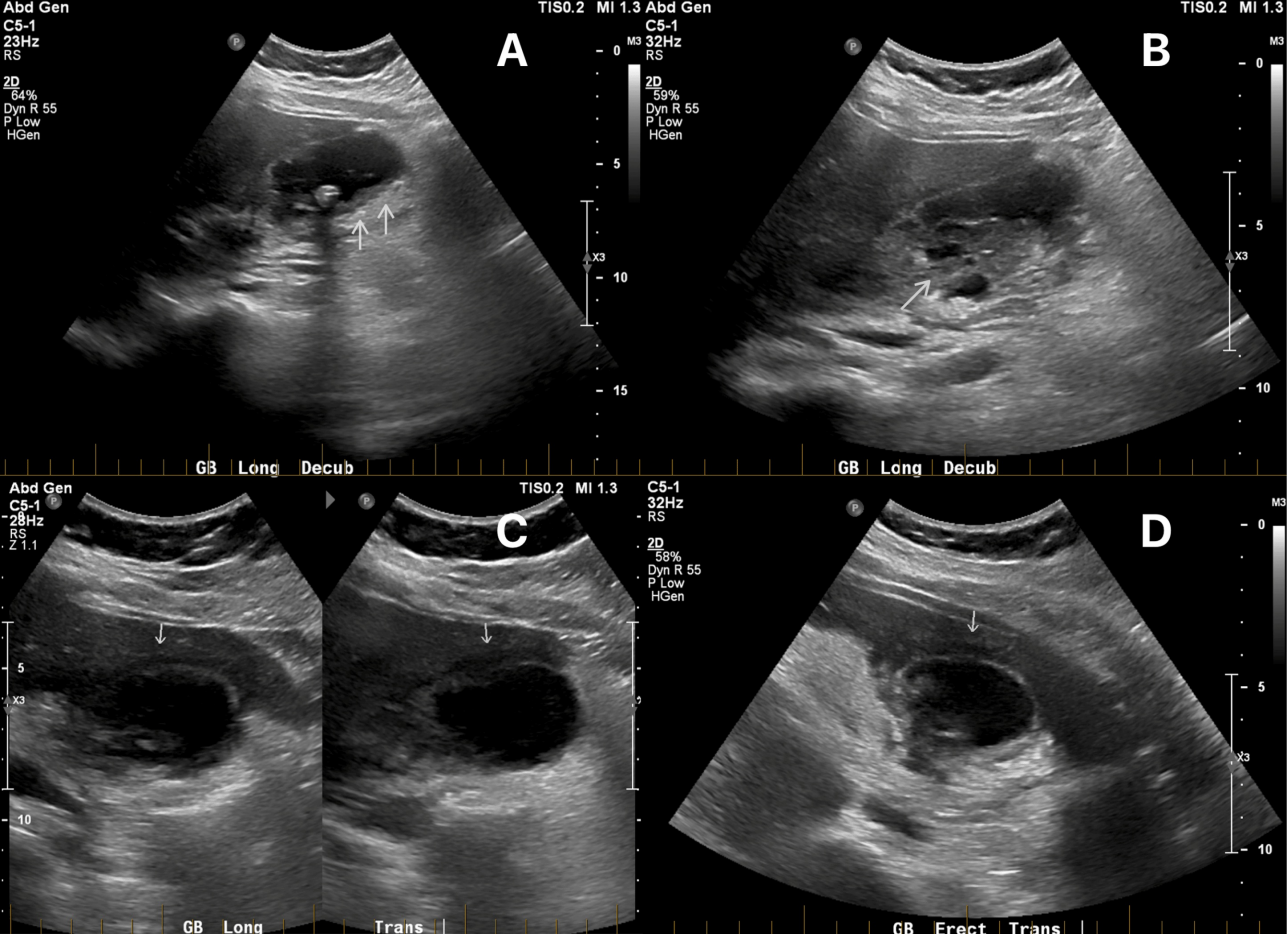
Ultrasound of the gallbladder on the second presentation. While initially reported as typical acute calculous cholecystitis with a thickened gallbladder wall of 3.78 mm, features of XGC are noted in retrospect. The multiple hypodense nodules occupying a large area of the thickened gallbladder wall are suggestive of XGC (white arrows). Also seen is a calculus within the gallbladder body (A).

She presented to the emergency department 17 days later with vague, generalized abdominal pain, subjective fevers, and lethargy. There was mild tenderness to palpation in the right upper quadrant. Her blood tests again displayed elevated inflammatory markers but normal liver function tests (Table 1). A repeat ultrasound of the gallbladder was reported with features of typical acute calculous cholecystitis, evidenced by a thickened gallbladder wall of 3.78 mm and multiple gallstones (Figure 2). Features of XGC are present in retrospect (Figure 2); however, these were not noted by the treating team or reporting radiologist at the time. Laparoscopic cholecystectomy was planned for three days’ time, and she was given intravenous ampicillin and gentamicin in the interim.

**Figure 3:**
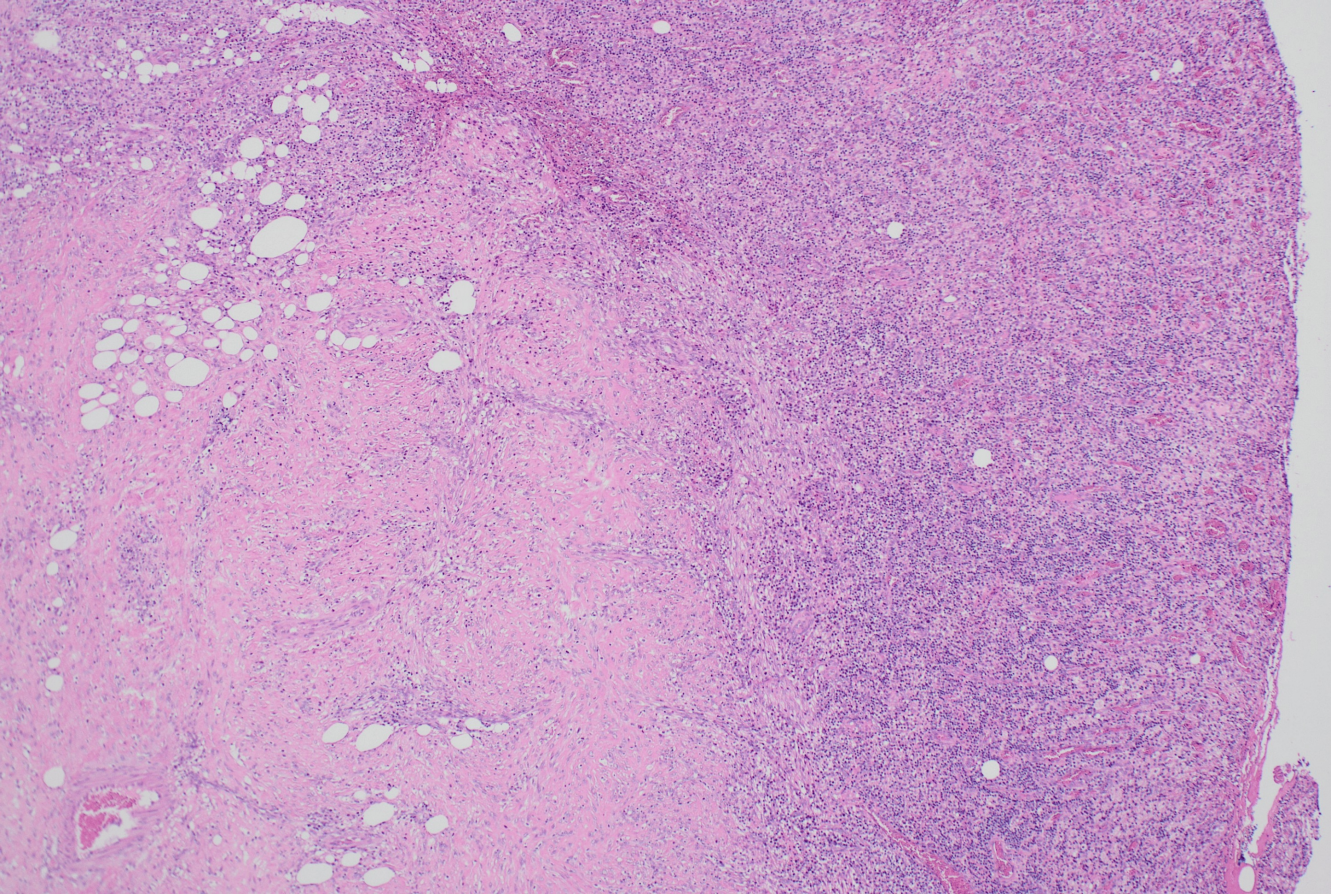
Florid transmural inflammation with an ulcerated appearance.

**Figure 4:**
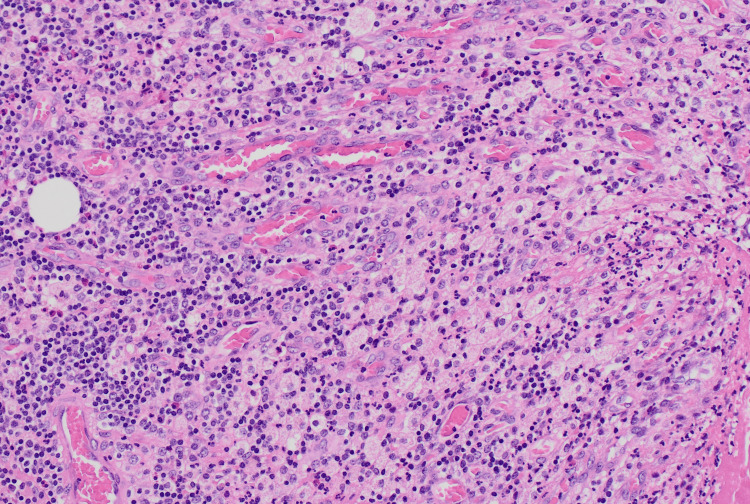
Prominent collections of foamy histiocytes in the background of mixed acute-on-chronic inflammation.

A standard laparoscopic cholecystectomy setup was employed, utilizing a 12 mm Hasson infra-umbilical entry, an 11 mm epigastric port, and two 5 mm ports in the right upper quadrant, with high-flow capnoperitoneum maintained at a target pressure of 12 mmHg. The cholecystectomy was difficult and complicated by apparent severe chronic inflammation. The gallbladder wall exhibited segmental necrosis with pseudo-tumorous incorporation into adjacent structures. The omentum was densely adherent to the fundus, the duodenum to the medial neck, and the transverse colon to the anterior neck, rendering the tissue planes indistinguishable and significantly complicating dissection. Intraoperative images were not taken at the time as the presentation mimicked that of severe acute-on-chronic cholecystitis, with XGC only confirmed through postoperative histological analysis. A critical view of safety was unable to be dissected given the fusion of structures in the hepatocystic triangle. Accordingly, a subtotal cholecystectomy was performed. The fundal wall that could be separated from other structures was removed and a washout was conducted. Multiple small cholesterol stones, each approximately 10 mm in diameter, were removed. Given the severe inflammation, the cystic stump was too friable to close securely. Accordingly, a Blake drain was placed in the remnant neck of the gallbladder. As anticipated, high-volume bile leakage was observed through the drain, with outputs of 490 mL and 400 mL recorded at 10 and 21 hours postoperatively, respectively. To address this, endoscopic retrograde cholangiopancreatography (ERCP) was performed on postoperative day 2 with the aim of decompressing the cystic duct by placing a stent in the common bile duct. A 7-French double-flange plastic stent was successfully inserted. Over the next eight days, the biliary drain output steadily decreased and became more serous, at which point the drain was removed. The patient was discharged home following a 14-day admission. At this time, histopathology results for the gallbladder specimen were reported, indicating a markedly thickened gallbladder wall due to muscle hypertrophy, fibrosis, and mixed acute-on-chronic inflammation (Figure 3). The mucosa was replaced by florid inflammation, with no viable mucosa identified. Prominent collections of foamy histiocytes were noted throughout the gallbladder wall (Figure 4). A histological diagnosis of XGC was made. At one-month follow-up, the patient was well and symptom-free with no new issues. Two months postoperatively, she underwent an elective ERCP for stent removal. The procedure was completed without complications, and the patient was discharged home the same afternoon.

## Discussion

XGC is a rare chronic inflammatory condition of the gallbladder wall characterized by a diffuse, destructive inflammatory process, proliferative fibrosis, and the infiltration of lipid-laden macrophages and foamy histiocytes [3]. It has been sparsely described in the literature and is poorly understood, with an estimated incidence rate of approximately 1.46% based on cholecystectomy samples evaluated from a sample of 12,426 [8]. Male predominance has been reported with a male-to-female ratio of 2:1 [8]. XGC is most commonly seen in adults over the age of 50 [6], and its occurrence in the pediatric age group is exceptionally rare, with very few reports in the literature documenting its occurrence in children or adolescents. 

In pediatric patients, the exact pathogenesis of XGC remains uncertain, with the currently accepted hypothesis in adults suggesting it could result from the combination of biliary outflow obstruction and gallbladder inflammation. It is thought that obstruction of bile outflow, whether by gallstones or another factor, allows bile to penetrate the gallbladder stroma through the Rokitansky-Aschoff sinuses. Accumulated histiocytes phagocytize the bile leading to xanthogranuloma formation in the gallbladder wall, inducing a chronic inflammatory state and subsequently XGC [4]. Cholelithiasis is present in 69-96% of cases and obstructs the cystic duct in 80% of cases, supporting the theory that biliary obstruction is related to the onset of the disease [6,9]. In the present case, gallstones were noted to be impacted in the neck of the gallbladder on ultrasound (Figure 1); however, there were no biochemical features of complete biliary obstruction (Table 1), suggesting that XGC may develop with just partial biliary outflow obstruction. 

Compared to typical acute calculous cholecystitis, XGC raises unique diagnostic and management challenges, especially in the pediatric population. XGC can take on a pseudo-tumoral aspect, forming an inflammatory mass within the gallbladder wall that can invade adjacent organs such as the liver, omentum, duodenum, and colon, mimicking neoplastic extension [4,10]. In this case, the necessity for a subtotal cholecystectomy underscores the complexity of managing XGC where complete resection was not feasible without significant risk to surrounding organs. Achieving a critical view of safety was hindered by the xanthogranulomatous infiltration into the adjacent structures, significantly altering the anatomy. Subtotal cholecystectomy has been described as a safe damage control procedure in such scenarios, effectively addressing inflammation while minimizing the risk of injury to surrounding structures [11]. Despite this, the perioperative morbidity and risk profile associated with a subtotal cholecystectomy is significantly higher than that of a total laparoscopic cholecystectomy [12], as evidenced in this case by the prolonged hospital admission and need for ERCP. Complete cholecystectomy is often not possible with XGC due to poor visualization of the cystohepatic triangle, and the length of hospital admission is generally longer with complication rates of up to 20% for these patients [9].

A challenging cholecystectomy in pediatric patients carries risks of potentially severe complications, as in adults. In this case, a preoperative diagnosis of XGC, including its pseudo-tumorous invasion into adjacent structures, could have significantly minimized complications. Early recognition would have prompted advanced imaging, such as magnetic resonance imaging, to better evaluate anatomy and anticipate a challenging cholecystectomy. This information could facilitate a safer, more streamlined surgical plan, reducing operative time and mitigating the risk of iatrogenic injuries, such as bile duct damage from unnecessary dissection attempts. Additionally, early identification of XGC allows for comprehensive preoperative counseling of patients and families, setting realistic expectations about potential complications, prolonged recovery, and the likelihood of advanced interventions such as subtotal cholecystectomy or ERCP. However, XGC can be a diagnostic dilemma, and diagnosing this entity on imaging alone can be extremely challenging. A recent retrospective cohort study of 100 patients with histologically confirmed XGC noted that none of the patients were prediagnosed with the condition prior to their surgery [7]. This was despite ultrasonography of the gallbladder being performed in all 100 patients preoperatively, with some selectively being investigated with computed tomography (CT) or magnetic resonance imaging as well. Although the preoperative radiological diagnosis of XGC is difficult, the presence of intramural hypoechoic nodules on sonography or hypodense bands on CT is considered to be a characteristic feature that is highly suggestive of XGC [7,13]. On ultrasound, xanthogranulomatous nodules present well-defined hypoechoic regions within the thickened gallbladder wall [3]. Such nodules on ultrasonography have been observed in 35% and 73% of cases by Parra et al. [14] and Kim et al. [15], respectively. In retrospect, this can be seen on the patient’s second ultrasound (Figure 2); however, its significance was not noted by the treating team nor was it reported by the radiologist at the time. Intramural hypodense nodules are also one of the most specific findings of XGC on CT, with approximately a third of patients exhibiting this feature [7,14].

## Conclusions

XGC is exceedingly rare in the pediatric population with very few documented cases in the literature. This case offers valuable insight into the diagnostic and surgical complexities associated with this rare condition and underscores the need for heightened awareness of XGC as a differential diagnosis in pediatric gallbladder disease. While the preoperative diagnosis of XGC is difficult, key imaging findings such as intramural hypodense nodules on ultrasonography or hypodense bands on CT are highly suggestive of the disease. This article underscores the importance of heightened clinical and radiological awareness in identifying this condition early, particularly in atypical patient populations like children. Early recognition of XGC allows for meticulous preoperative planning to mitigate complications arising from the inflammatory and pseudo-tumorous features characteristic of XGC. However, further research is required to elucidate the pathogenesis of XGC in pediatric patients and refine diagnostic and therapeutic strategies.
